# Managing Severe Dysgeusia and Dysosmia in Lung Cancer Patients: A Systematic Scoping Review

**DOI:** 10.3389/fonc.2021.774081

**Published:** 2021-11-22

**Authors:** Ana Sofia Spencer, David da Silva Dias, Manuel Luís Capelas, Francisco Pimentel, Teresa Santos, Pedro Miguel Neves, Antti Mäkitie, Paula Ravasco

**Affiliations:** ^1^ Department of Medical Oncology, Centro Hospitalar Universitário de Lisboa Central, Hospital de Santo António dos Capuchos, Lisbon, Portugal; ^2^ Department of Medical Oncology, Centro Hospitalar Universitário do Algarve, Hospital de Faro, Faro, Portugal; ^3^ Centre for Interdisciplinary Research in Health of Universidade Católica Portuguesa (UCP), Lisbon, Portugal; ^4^ CatolicaMed Platform of of Universidade Católica Portuguesa (UCP), Lisbon, Portugal; ^5^ Universidade Católica Portuguesa (UCP), Institute of Health Sciences, Centre for Interdisciplinary Research in Health (CIIS), Lisbon, Portugal; ^6^ BlueClinical, Matosinhos, Portugal; ^7^ European University, Lisbon, Portugal; ^8^ Católica Medical School, Universidade Católica Portuguesa (UCP), Lisbon, Portugal; ^9^ Department of Otorhinolaryngology-Head and Neck Surgery, Helsinki University Hospital and University of Helsinki, Helsinki, Finland; ^10^ Research Program in Systems Oncology, Faculty of Medicine, University of Helsinki, Helsinki, Finland; ^11^ Division of Ear, Nose and Throat Diseases, Department of Clinical Sciences, Intervention and Technology, Karolinska Institute and Karolinska University Hospital, Stockholm, Sweden; ^12^ Centro de Investigação Interdisciplinar Egas Moniz (CiiEM), Instituto Universitário Egas Moniz (IUEM), Quinta da Granja, Monte de Caparica, Caparica, Portugal

**Keywords:** dysgeusia, dysosmia, taste and smell alterations (TSAs), lung cancer, dietary counselling, zinc, weight loss

## Abstract

**Introduction:**

Lung cancer (LC) is highly prevalent worldwide, with elevated mortality. In this population, taste and smell alterations (TSAs) are frequent but overlooked symptoms. The absence of effective therapeutic strategies and evidence-based guidelines constrain TSAs’ early recognition, prevention and treatment (Tx), promoting cancer-related malnutrition and jeopardizing survival outcomes and quality of life.

**Objectives:**

To systematically review the literature on TSAs in LC patients, understand the physiopathology, identify potential preventive and Tx strategies and to further encourage research in this area.

**Methods:**

Literature search on English language articles indexed to PubMed, CINALH, SCOPUS and Web of Science using MeSH terms “Lung neoplasms”,”Dysgeusia”, “Olfaction Disorders”, “Carcinoma, Small Cell”,”Carcinoma, Non- Small-Cell Lung “Adenocarcinoma of Lung”,”Carcinoma, Large Cell”, and non-MeSH terms “Parageusia”, “Altered Taste”, “Smell Disorder”, “Paraosmia”, “Dysosmia”,”Lung Cancer” and “Oat Cell Carcinoma”.

**Results:**

Thirty-four articles were reviewed. TSAs may follow the diagnosis of LC or develop during cancer Tx. The estimated prevalence of self-reported dysgeusia is 35-38% in treatment-naïve LC patients, and 35-69% in those undergoing Tx, based on studies involving LC patients only.

One prospective pilot trial and 1 RCT demonstrated a clinically significant benefit in combining flavor enhancement, smell and taste training and individualized nutritional counselling; a systematic review, 1 RCT and 1 retrospective study favored using intravenous or oral zinc-based solutions (150mg 2-3 times a day) for the prevention and Tx of chemotherapy (CT) and radiotherapy (RT) -induced mucositis and subsequent dysgeusia.

**Conclusions:**

This is the first review on dysgeusia and dysosmia in LC patients to our knowledge. We propose combining taste and smell training, personalized dietary counselling and flavor enhancement with oral zinc-based solutions (150mg, 2-3 times a day) during CT and/or RT in this population, in order to prevent and help ameliorate Tx-induced dysgeusia and mucositis. However due to study heterogeneity, the results should be interpreted with caution. Developing standardized TSA measurement tools and performing prospective randomized controlled trials to evaluate their effect are warranted.

## Introduction

The second most frequently occurring cancer globally is lung cancer, with an incidence of 2,206.77 (excluding non-melanoma skin cancers), only surpassed by breast cancer. Also, it has the highest mortality of any cancer, accounting for 1,796,144 deaths in 2020 (1).

Whilst many symptoms are associated with lung cancer, clinicians frequently observe taste and smell alterations (TSAs). TSAs can be present upon initial diagnosis or develop during the course of cancer treatment, and can lead to a change in food preferences, resulting in reduced nutrient intake, a higher probability of weight loss and impact on patient’s health-related quality of life (HRQoL) through a decrease in the pleasure of eating (2).

Although taste and smell belong to anatomically distinct systems, they are intimately connected in the sensory perception of food (3).

The chemical interplay between taste and smell senses is critical in helping humans gather information about themselves and their surrounding environment, from aiding social interaction, detecting potential dangers and the enjoyment of food consumption (4).

When consuming food or drinks, saliva helps dissolve tastant molecules facilitating their interaction with taste receptors in the mouth, leading to the activation of subsequent cascades that send signals to the brain through specialized nerve cells. The signals generated by the five basic tastes – bitter, salty, sour, sweet and umami - interact with other signals triggered by eating and drinking, such as smell and the trigeminal sensations of irritancy, temperature and texture. Together, these combine to create the sensations associated with flavor. Upon stimulation, taste bud cells trigger the activation of proximal gustatory afferent fibers that convey signals, *via* the facial (VII), glossopharyngeal (IX) and vagal (X) nerves, to the rostral division of the solitary tract nucleus in the brain stem. Additional taste neurons of a higher-order project these signals to the thalamus and subsequently to the gustatory cortex. The taste signals are then projected to a variety of brain structures *via* neurons in the gustatory cortex, including the mid-brain dopaminergic regions, amygdala, and orbitofrontal cortex. The orbitofrontal cortex is also targeted by neurons involved in olfaction and oral mouth-feel which is thought to be central in perceiving flavour (5).

The axons of olfactory sensory neurons project signals to the olfactory bulb. The piriform cortex is the main recipient of afferents from the olfactory bulb. The axons of neurons projected from the olfactory bulb are broadly dispersed across the surface of the piriform cortex, and individual piriform cortex neurons respond to numerous, chemically distinct odorants, clustering odor representations and enabling them to preferentially signify odor relationships (6).

The olfactory and taste systems can be damaged in multiple ways that reduce their function, including age, bacterial and viral illnesses, trauma, surgical damage, severe allergies, chronic rhinosinusitis, inborn genetic disorders, neurological diseases, some medications and cancer treatments (4).

Both quantitative and qualitative changes can occur as a result of taste disorders. Quantitative changes include ageusia (total taste loss), hypogeusia (partial taste loss) and hypergeusia (increased taste responsiveness). Qualitative changes include phantogeusia (the sensation of taste with stimuli absent, also known as “oral phantoms”) and dysgeusia (the persistence in the mouth of slaty, bitter, rancid or metallic taste sensations after finishing a meal (3, 5).

Typically, smell disorders are divided into four categories, depending on their odor perception impact. Firstly, the absence of smell perception, or Anosmia. Secondly, a quantitatively reduced ability to perceive smells, or Hyposmia. Thirdly, a qualitative distortion of the normally perceived smell, or Parosmia. And finally, the perception of smells in the absence of an odor, or Phantosmia (4).

TSAs can be evaluated through quantitative analysis, chemical stimuli or surveys (6). As described above, the nature of the chemical senses is incredibly complex and interconnected, therefore being important to assess smell and taste together (7).

However, a limiting factor to the evaluation of TSAs arises from the heterogeneity of the systems used to measure them and the fact that these symptoms are often underestimated and overlooked, probably due to their non-life-threatening nature (8).

Dysgeusia affects 46-77% of patients with cancer, from roughly 53% of patients receiving treatment with chemotherapeutic drugs, to 66% of those receiving radiotherapy (RT) and 76% of those patients prescribed both treatments (2, 8–11).

One review reported a prevalence of 12-84% of self-reported taste problems among cancer patients (12).

Another review of patients receiving chemotherapy (CT) reported a prevalence of alterations to taste of 45-84% and of alterations to smell of 5-60%. It also found that in patients with advanced cancer, almost 80% reported TSAs (13).

One unicentric study involving 239 patients with different cancer types undergoing cancer treatment (including nine patients with lung cancer) reported a 54% rate of dysphagia, 62% rate of taste changes and 35% rate of smell changes (14).

These wide ranges relate to study heterogeneity and the absence of standardized questionnaires and methodologies for patients to self-report the presence of chemosensory alterations (3, 12).

Malnutrition is a common finding in cancer patients, with an incidence varying between 31–87%. Undernutrition and weight loss may result from reduced energy intake, increased energy requirements, impaired nutrient absorption, tumor-related catabolism and inflammation leading to muscle wasting, anticancer treatment side effects and patient’s poor psychological state (15).

A study by Joseph P.V. et al. performed with 1,329 cancer patients of various types undergoing CT, concluded that patients reporting treatment-related taste alterations suffered significantly from neuropsychological symptoms. These included higher levels of depression, anxiety, sleep disturbance and fatigue when compared to patients with no change in taste (16).

In line with the psychosocial impact of CT, Sasaki et al. evaluated the perception of symptoms through a 94-item questionnaire in 49 patients receiving chemotherapy in a hospital in Japan. It was concluded that the most frequent and troublesome non-physical concern of patients was the fact that “it affected their families or partner” (17).

Multiple studies have highlighted that poor prognosis and quality of life (QoL) in cancer patients is intimately associated with weight loss, which in turn raises the probability of adverse side-effects from treatment and impairing the response of a tumor to therapy (15).

This is reflected by the fact that malnutrition, and not malignancy, is responsible for 20% of cancer patients deaths, meaning it is essential for patients to maintain an appropriate dietary intake throughout their cancer treatment (18).

This scoping review aims to comprehensively understand the pathophysiology, impact, prevention, and treatment of TSAs in lung cancer patients, and to identify gaps in our current scientific knowledge so as to encourage further research in those areas. A preliminary search on Pubmed and the Cochrane Database of Systematic Reviews was conducted and no existing or in progress systematic reviews on this topic were identified.

## Methods

### Inclusion Criteria

This scoping review was built on the basis of “The Joanna Briggs Institute” methodology. The inclusion criteria for this review were based upon the PCC elements (Population, Concept and Context). This review examined all studies and reports focusing on TSAs occurring in adult lung cancer patients, either being treatment-naïve or submitted to cancer treatment, even if the studies were not limited to this specific cancer type. Studies focusing on the pathophysiology, management and treatment approaches of TSAs were also considered.

### Types of Sources

This scoping review considered experimental and quasi-experimental study designs, including randomized controlled trials, non-randomized controlled trials, before and after studies and interrupted time-series studies. Analytical observational studies, including prospective and retrospective cohort studies and analytical cross-sectional studies; and descriptive observational study designs including case series, individual case reports and descriptive cross-sectional studies, were considered for inclusion. Text and opinion papers that met the inclusion criteria were also considered.

### Search Strategy

A literature search was performed in July 2021 including English language papers indexed to Pubmed, CINALH, SCOPUS and Web of Science, using the MeSH terms “Dysgeusia”, “Olfaction Disorders”, “Lung neoplasms”, “Carcinoma, Non-Small-Cell Lung”, “Carcinoma, Small Cell”, “Adenocarcinoma of Lung”, “Carcinoma, Large Cell”, and the additional search terms “Altered Taste”; “Parageusia”, “Smell Disorder”, “Dysosmia”, “Paraosmia”, “Lung Cancer” and “Oat Cell Carcinoma”, with no date range filter (**Table 1**). Articles from other sources were also searched to complement the review. Additionally, bibliography lists of all retrieved articles were searched for relevant studies.

**Table 1:** Summary of the search strategy.

Search Strategy	
**Pubmed**	[(((((((((((Taste Disorders[MeSH Terms)] OR Taste Disorders[Title/Abstract]) OR Dysgeusia[MeSH Terms]) OR Dysgeusia[Title/Abstract]) OR Olfaction Disorders[MeSH Terms]) OR Olfaction Disorders[Title/Abstract]) OR Altered Taste[Title/Abstract]) OR Parageusia[Title/Abstract]) OR Smell Disorder[Title/Abstract]) OR Dysosmia[Title/Abstract]) OR Paraosmia[Title/Abstract])) AND ((((((((((((Carcinoma, Large Cell[MeSH Terms]) OR Carcinoma, Large Cell[Title/Abstract]) OR Adenocarcinoma of Lung[MeSH Terms]) OR Adenocarcinoma of Lung[Title/Abstract]) OR Carcinoma, Small Cell[MeSH Terms]) OR Carcinoma, Small Cell[Title/Abstract]) OR Carcinoma, Non-Small-Cell Lung[MeSH Terms]) OR Carcinoma, Non-Small-Cell Lung[Title/Abstract]) OR Lung neoplasms[MeSH Terms]) OR Lung neoplasms[Title/Abstract]) OR Lung Cancer[Title/Abstract]) OR Oat Cell Carcinoma[Title/Abstract])
**CINAHL**	(MM Lung neoplasms OR TI Lung neoplasms OR AB Lung neoplasms OR MM Carcinoma, non-small cell lung OR TI Carcinoma, non-small cell lung OR AB Carcinoma, non-small cell lung OR MM Carcinoma, Small Cell OR TI Carcinoma, Small Cell OR AB Carcinoma, Small Cell OR MM Adenocarcinoma of Lung OR TI Adenocarcinoma of Lung OR AB Adenocarcinoma of Lung OR TI Carcinoma, Large Cell OR AB Carcinoma, Large Cell OR TI Lung Cancer OR AB Lung Cancer OR TI Oat Cell Carcinoma OR AB Oat Cell Carcinoma) AND (MM Dysgeusia OR TI Dysgeusia OR AB Dysgeusia OR MM Olfaction disorders OR TI Olfaction disorders OR AB Olfaction disorders OR MM Taste Disorders OR TI Taste Disorders OR AB Taste Disorders OR TI Altered Taste OR AB altered states of consciousness OR TI Parageusia OR AB Parageusia OR TI Smell Disorder OR AB Smell Disorder OR TI Dysosmia OR AB Dysosmia OR TI Paraosmia OR AB Paraosmia)
**Web Of Science**	Search similar to the two search engines above
**SCOPUS**	Search similar to the two search engines above

### Data Extraction and Presentation

Data were extracted from papers by two authors independently, using the Rayyan data extraction tool. The data extracted included specific details about TSAs in cancer patients in general and in lung cancer patients, as well as key relevant information to the review questions. Where there was disagreement between authors, the issues were discussed to reach a consensus. Following extraction, all full texts were subsequently independently screened by the reviewers. The extracted data are presented in tabular form, to align with the objectives of this scoping review. We attached a narrative summary to accompany the tabulated results to describe the relationship between the results and the review’s objective and questions.

## Results

### Study Inclusion

**Figure 1:**
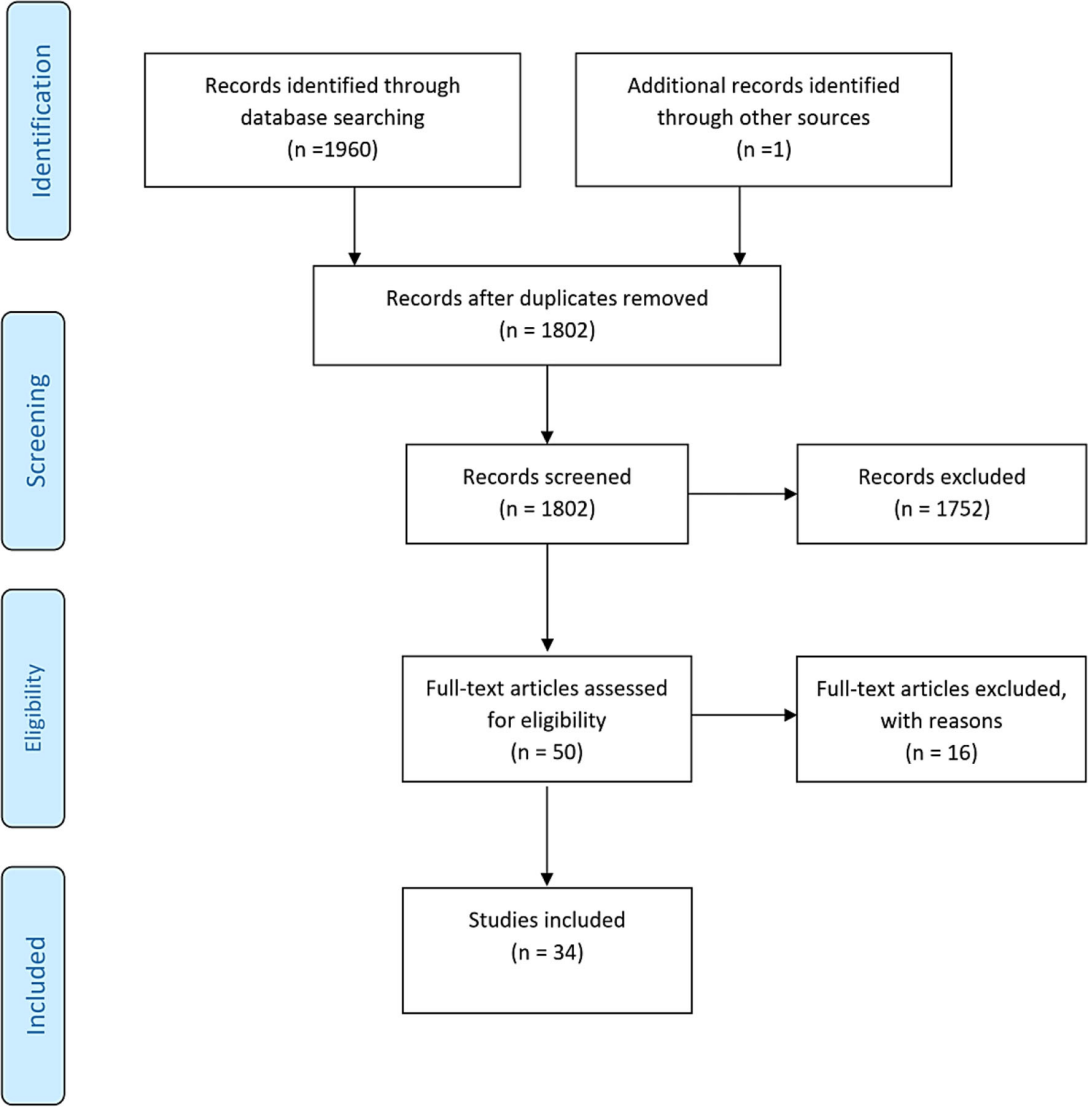
PRISMA flowchart outlining the process for selecting the included articles.

A total of 1,960 citations were identified. After duplicate removal (n=159), 1,802 titles and abstracts meeting the inclusion criteria remained for analysis. 1,752 references were then excluded, with 50 full texts retrieved for analysis. Sixteen studies were excluded, leaving 34 articles to be included in this review. The PRISMA flowchart (**Figure 1**) describes the flow of decisions of this process. **Table 5** records studies ineligible following the full text review.

### Review findings

#### Factors Influencing TSAs

The exact underlying mechanisms behind chemosensory alterations in cancer patients is not fully known, due to odor and taste abnormalities having a multifactorial etiology, heterogeneous cancer population studies, variability of the definition of “taste” and on the endpoints used in the studies, as well as the lack of standardized measurement tools to evaluate TSAs (3, 12).

A study by Belqaid et al. demonstrated that the intensity of TSAs tends to change over time within the course of lung cancer treatment. Additionally, TSAs are influenced by the presence of additional symptoms, side-effects of treatment and other individual and contextual factors (19).

Additional factors can also contribute to reduced smell and taste perception, including, poor oral hygiene, lack of saliva and alcohol or nicotine abuse (20).

Eating-related symptoms such as nausea, dry mouth, premature satiety, appetite loss and fatigue also interrelate with TSAs (21).

Xerostomia is highly related to taste alteration, as food particles stimulate taste bud taste receptor cells within the lingual papillae, when in solution. Therefore, reduced secretion and increased saliva viscosity may interfere with flavor molecules being transported to taste and olfactory receptors (3).

In addition, changes in cell volume or osmolar content of any of the central nervous system neurons implicated in the sense of taste are influenced by extracellular hypo-osmolality and may potentially inhibit the sense of taste (22).

In a systematic review by Nolden et al., the majority of the reviewed articles identified no significant relationship between measures of smell or taste and intake of food and enjoyment. However, it was suggested that where lower taste sensitivity is experienced by patients (higher detection thresholds), incidences of food avoidance also increase. The most common taste alterations were observed for sweet and, to some extent, bitter perception, whereas alterations in cancer patients to salt and sour perception where less frequent. The alterations were associated with lower consumption, appetite, and patients avoiding certain foods (12).

Williams and Cohen compared the taste threshold levels of 30 male lung cancer subjects before the start of RT or CT with a healthy control group, demonstrating a significant reduction in the sensitivity for sour in the lung cancer group. No significant differences were noted concerning sweet, bitter or salty tastes, although there were individuals with recognition levels that differed considerably from controls. It was concluded that diet therapy management for lung cancer patients should be individualized, in order to maintain the diary amount of protein and calories (23).

Turcott et al. evaluated changes in the thresholds for detecting and recognizing sweet, bitter and umami tastes in patients with non-small cell lung cancer (NSCLC) receiving CT treatments of cisplatin and paclitaxel-based, as well as their association with nutritional and HRQoL parameters. It was concluded that taste buds’ impairment and disturbance of renewal cell processes might increase the detection thresholds for sweet, sour, salty, bitter or umami. On the other hand, higher sensitivity to umami recognition (hypergeusia) had significant association with a global HRQoL status deterioration and loss of appetite (p=0.016 and 0.115 respectively) (24).

McGreevy et al. investigated the characteristics of severe TSAs reported by 89 lung cancer patients undergoing CT. Patients reporting TSAs were younger and more frequently smokers. Gender was a statistically significant variable, with higher numbers of women reporting TSAs (25).

On the other hand, Yoshimoto et al. investigated CT impact on the smell and taste of 35 Japanese patients with lung cancer through the use of questionnaires, identifying no statistically significant associations between smell alterations and age, gender or smoking history. Patients became more sensitive to sweet and salty tastes, but less so for umami and bitter. This might have been influenced by patients’ psychological stress, oral hygiene and smoking status, as well as the study’s small sample size (11).

Amézaga et al. also found no statistically significant differences in taste and smell alterations between older and younger patients (3).

Zabernigg et al. found that elderly patients or patients with nicotine abuse reported fewer TAs, which might be explained by the fact that these two groups already tend to suffer from hypogeusia of some degree, making CT-induced changes less noticeable (10).

#### Taste and Smell Alterations in Treatment- Naïve Lung Cancer Patients

**Table 2** summarizes the existing evidence on TSAs in treatment-naïve lung cancer patients, included in the review. Turcott et al. found a 35% prevalence of self-reported dysgeusia among 65 treatment-naïve non-small cell lung cancer patients submitted to self-reporting taste and smell questionnaires and a rinse stimuli technique. A minimal concentration of taste stimuli was required for most patients to perceive the stimuli; however they commonly could not recognize the taste. Patients with dysgeusia presented with a significantly reduced lean-body mass (p=0.027), a significant increase in fat mass (p=0.027) and gastrointestinal symptoms, including nausea (p=0.042), anorexia (p=0.004), early satiety (p<0.0001) and reduced food consumption (p=0.01). They also had clinically significant alterations in HRQoL scales (2).

**Table 2:** Summary of evidence concerning treatment-naïve lung cancer patients included in the review.

Authors	Year	Type of Study	Sample Size	Variables Assessed	Method used to evaluate TSAs	Main Results
**Turcott et al. (** 2)	2020	Cross-sectional, unicentric	N= 65 treatment-naïve non-small-cell lung cancer (SCLC) pts	-Dysgeusia -HRQL -Nutritional Status	Self-reporting taste Questionnaire	-35% prevalence of self-reported dysgeusia -Pts with dysgeusia had less lean body mass (p=0.027); higher fat mass (p=0.027); nausea (p=0.042); anorexia (p=0.004); early satiety (p<0.0001); clinically significant alterations in HRQL
**Belqaid et al. (** 13 **)**	2014	Prospective, observational, unicentric	N=215 pts under investigation for lung cancer, of which N=117 were diagnosed with lung cancer	-TSAs -Nutritional Status	Taste and Smell Survey	-38% prevalence of TSAs in pts with and without lung cancer; generally mild - Pts with lung cancer reporting TSAs had higher frequency of weight loss ≥10% (p<0.05)
**Williams & Cohen (** 23 **)**	1978	Prospective, observational, unicentric	N=60; 30 male pts with lung cancer and 30 male healthy controls	- Taste Acuity	Method of Henkin	-Lung cancer pts had lower sensitivity for sour (p=0.05)
**Singh et al. (** 30 **)**	2009	Case Report	–	–	–	- Case report of concomitant dysgeusia, hyponatremia and SCLC. - Dysgeusia is rarely seen with SIAD; various reports on its association with lung cancer, mostly with SCLC histology.
**Nakazato et al. (** 27 **)**	2006	Case Report	–	–	–	- Case report of dysgeusia (sweet taste of nearly all food) and hyponatremia related to SIAD, found to be associated with large-cell lung carcinoma. -Dysgeusia might have been caused by the tumor producing an unknown taste modifying substance, leading to structural changes in the taste receptor membrane when the extracellular sodium levels decreased. Miraculin could be a potential candidate substance.
**Karthik et al. (** 29 **)**	2004	Case Report	–	–	–	- Case report of dysgeusia as paraneoplastic syndrome related with lung adenocarcinoma.
**Croghan et al. (** 28 **)**	2003	Case Report	–	–	–	-Case report of dysgeusia (constant sweet taste sensation) and hyponatremia, found to be associated with a diagnosis of SCLC.
**Ishimaru et al. (** 26 **)**	1999	Case Report	–	–	–	-Case report of a lung cancer metastasized to the right frontal lobe, causing compression of the olfactory sulcus. The average olfaction recognition threshold improved after craniotomy.
**Panayiotou et al. (** 22 **)**	1995	Case series (3 case reports)	–	–	–	-Changes in the extracellular sodium concentration may modulate the sweet receptor. - The onset of persistent dysgeusia (especially if unpleasant sweet taste) should prompt measurement of serum sodium concentration and the consideration of lung carcinoma.
**Kamoi et al. (** 39 **)**	1987	Case Report	–	–	–	- Case report of SCLC associated with hyponatremia, renal sodium loss and inappropriate antidiuresis, due to increased secretion of atrial natriuretic peptide (ANP) by the atrial tissue. The natriuretic activity led to the glomerular filtration rate increasing and a decrease in tubular sodium resorption, increasing the renal fractional excretion of sodium.

Pts, patients; SIAD, Syndrome of Inappropriate Antidiuresis; SCLC, Small Cell Lung Cancer; TSAs, Taste and Smell Alterations.

In another study, Turcott et al. found self-reported dysgeusia was prevalent in 37.5% of NSCLC treatment-naïve patients, and a 34.5% prevalence post-CT, revealing a drastically high rate of taste disorders prior to CT, comparing to that of the general population (0.07-1.7%) - meaning that dysgeusia could be caused by the disease itself (24).

An observational study by Belqaid et al. involving 215 patients under investigation for lung cancer, found a self-reported TSA prevalence of 38% in the group of patients diagnosed with lung cancer (n=117), and symptoms were in general mild (13).

Ishimaru et al. described a case report of reversible hyposmia, after removal of a right frontal lobe lung cancer metastasis which was causing slight compression and swelling of the olfactory area in the brain (26).

Also, a triad of taste distortion with prominent sweet taste, hyponatremia and lung cancer diagnosis was firstly reported in a case series by Panayiotou et al., in 1995 (22).

Since then, few other reports have described the diagnosis of lung cancer in the context of dysgeusia with unpleasant sweet taste, with concomitant hyponatremia as the sole biochemical anomaly (27–30).

It is necessary to consider the lung cancer induced syndrome of inappropriate antidiuresis (SIAD) when cryptogenic dysgeusia is identified, particularly if patients report an unpleasant sweet taste (27).

For SIAD to be diagnosed, the osmolality of the patient’s urine when the effective plasma osmolality is low must exceed 100 mOsm per kilogram of water. Additionally, it is essential for clinical euvolemia to be present. Eliminating the underlying cause is the only definitive treatment for SIAD. For the majority of SIAD cases caused by malignant disease, effective antineoplastic treatment is typically the best course of action (31).

The most frequently implicated histological type is the small cell lung carcinoma. In all cases, dysgeusia seemed to disappear rapidly with normalization of serum sodium concentration (27–30).

One explanation for this is that extracellular sodium levels may modulate sweet receptors. Hyponatremia may decrease lingual sweetness receptor thresholds, although the sole cause of taste alteration cannot be completely attributed to a low sodium level alone. Nakazato et al. hypothesized that an unknown taste modifying substance could be produced by the tumor causing all foods to be interpreted by patients as sweet. This might occur due to structural changes in the taste receptor membrane when extracellular sodium levels decreased, allowing attachment of the taste modifier to sites on the sweet receptor (27). A candidate substance could be miraculin, a glycoprotein extracted from the West African berries *Richadella dulcifera*, which modifies taste through the alteration of taste receptor configuration (27, 30).

#### Taste and Smell Alterations in Lung Cancer Patients Undergoing Treatment

Multiple factors contribute to the risk of CT leading to toxic effects in the oral cavity, including the high renewal rates of oral tissues, damage to the mucosal microflora, salivary glands and development of neuropathy, where axonal degeneration of nerve conduction velocity occurs in up to 80% of cases affecting taste sensitivity and contributing to dysgeusia. The glossopharyngeal, facial and vagus nerves give rise to the corda tympani and greater petrosal nerves, all involved in the taste pathway (24).

**Table 3** summarizes the evidence on TSAs in lung cancer patients undergoing systemic treatment, included in the review. A study by Zabernigg et al. investigated the prevalence of taste alterations (TAs) in 197 cancer patients undergoing CT, of which 54.3% had lung cancer. Almost 70% of patients reported TAs at least once during the study period, with 17.6% reporting moderate to severe TAs (10).

**Table 3:** Summary of evidence concerning lung cancer patients undergoing systemic treatment included in the review.

Authors	Year	Type of Study	Sample Size	Variables Assessed	Method used to evaluate TSAs	Main Results
**Nolden et al. (** 12 **)**	2019	Systematic Scoping Review	11 studies including 578 participants (380 with cancer and 198 controls); all of the studies evaluated taste change and 5 also evaluated smell changes	- Taste changes (detection and recognition thresholds) for sweet, sour, bitter, salty and umami, and their relationship between food behavior in patients undergoing cancer treatment. - Smell changes (identification, sensitivity and discrimination) and their relationship between food behavior in patients undergoing cancer treatment.	- Whole mouth, filter paper disks and taste strips - Sniffin’ Sticks	- Cancer patients with appetite loss were more likely to prefer reduced sweetness levels; compared with patients without a reduced appetite. Effect sizes showed that sweet taste had the highest empirical evidence for food behavior involvement, with reduced appetite and overall lower energy intake. - The authors did not report any significant relationships between food behavior and olfactory measures.
**Yoshimoto et al. (** 11 **)**	2019	Cross-sectional, unicentric	N=35 Japanese lung cancer patients	- TSAs	-Self-reporting taste Questionnaire	- No significant associations between change in the sense of taste and CT cycles, age or BMI, were found. There was a trend towards an association with current smoking (p=0.083). - Patients reported a higher sensitivity to salty and sweet tastes and a lower sensitivity to umami after the start of CT. - No significant associations between change in the sense of smell and age, gender or smoking history were found. - Despite less favorable taste, Japanese patients did not change their dietary habits.
**Belqaid et al. (** 21 **)**	2018	Qualitative Interview Study	N=17 lung cancer patients; 13 women, 4 men	- Patients behavior while experiencing treatment-related TSAs	- Qualitative Interview	- TSAs implied coming to terms with the presence of these symptoms and finding new personal strategies to overcome them. - Health-care professionals’ involvement was generally described as limited. More normalizing information, emotional support and practical advice concerning dysgeusia could help better dealing with TSAs.
**Amézaga et al. (** 3 **)**	2018	Prospective, observational, unicentric	N=151 patients undergoing CT (13.2% with lung cancer)	- TSAs	- Interviewer-assisted TSAs’ Questionnaire	- Prevalence of 76% of taste disorders and 45% of smell alterations. - Anthracyclines, paclitaxel, docetaxel and carboplatin produced highest taste disturbance rates of all CT agents. - Xerostomia was the most frequent symptom reported, strongly associated with bad taste in mouth and taste loss. - Age did not significantly influence the occurrence of TSAs.
**Schalk et al. (** 40 **)**	2018	Cross-sectional, unicentric	N=138 patients; n= 42 with cancer (2.4% lung cancer patients), n= 57 with inflammatory disease, n=39 healthy controls	- Taste Acuity	- Tastant solutions through a “whole mouth method”	- Cancer patients had significantly increased detection thresholds for tastants sweet (p=0.024), salty (p=0.031) and umami (p=0.007) compared to healthy individuals; and for sweet (p=0.004) and sour (p=0.039), compared to patients with inflammatory disease. - No significant differences were found between treatment-naive cancer patients vs patients submitted to CT.
**Vigarios et al. (** 35 **)**	2017	Review	–	-Oral toxicities induced by targeted therapies and immune checkpoint inhibitors	–	- The incidence of mucositis was found to be between 8-20% with Erlotinib and between 17 to 24% with Gefitinib. - Crizotinib has been associated with moderate dysgeusia (grade 1-2) in 11-26% of treated patients. - The incidence of moderate dysgeusia (grade 1 or 2) with immune checkpoint inhibitors has been documented in 3% of PD-1 and PD-L1 treated patients. Xerostomia (generally grade 1-2) has been reported in about 6% of patients treated with Nivolumab and 4-7.2% of patients treated with Pembrolizumab.
**Ponticelli et al. (** 8 **)**	2016	Cross-sectional, unicentric	N=289 patients (8.7% with lung cancer)	- Taste Acuity - HRQL	- Taste Questionnaire	- Prevalence of dysgeusia during or after CT of 64%. - There was a statistically significant correlation between type of cancer and dysgeusia (p=0.012). - There was a statistically significant correlation between type of CT and occurrence of dysgeusia (p=0.031). - Patients with dysgeusia had a worse HRQL (p=0.002).
**Turcott et al. (** 24 **)**	2016	Cohort, unicentric	N=40 patients with lung cancer undergoing CT with Cisplatin/Paclitaxel	- Taste Acuity - Nutritional Status - HRQL	- Rinsing technique	- Prevalence of self-reported dysgeusia in treatment-naive lung cancer patients 37.5%; post-CT 34.5%. - CT induced a tendency for an increase in taste acuity for umami (p=0.109) and sweet (p=0.092), and an increase for bitter (p=0.02).
**Belqaid et al. (** 19 **)**	2016	Cohort, unicentric	N= 52 lung cancer patients under anti-cancer treatment	- Taste Acuity - Nutritional Status	- Taste and Smell Survey	- TSA characteristics changed over time, relative to the start of localized or systemic treatment. Patients’ experiences must be taken into account in order to adapt advice and support individual’s needs.
**McGreevy et al. (** 25 **)**	2014	Cohort (timepoint for data analysis ‘when TSAs were most severe’), unicentric	N= 89 patients under treatment for lung cancer	- TSAs - Nutritional Status	- Taste and Smell Survey	- 69% prevalence of TSAs after the start of cancer treatment -Patients reporting TSAs were on average more frequently smokers and younger -Women reported stronger TSAs -Patients with TSAs suffered more from loss of appetite, early satiety and nausea.
**Joussain et al. (** 32 **)**	2013	Cohort, unicentric	N=30 male patients; 15 patients with lung cancer; 15 controls	- Olfactory performance	- ETOC	- Cisplatin CT in lung cancer patients impaired the pleasure of perceived food odors (p<0.03), but not odor identification nor detection thresholds.
**Zabernigg et al. (** 10 **)**	2010	Cohort, unicentric	N= 197 cancer patients (54.3% with lung cancer) undergoing CT	- Taste Acuity - HRQL	- EORTC QLQ-C30 + 2 questions directed to TAs	- 69.9% of patients reported TAs in at least at one assessment time; 14.6% reported TAs in all assessment times. - 17.6% of patients reported moderate to severe TSAs. - TAs decreased significantly with age (p<0.001); - Patients with nicotine abuse reported less TAs (p=0.002) - Gender was not significantly associated with TAs. - TAs were significantly associated with appetite loss, fatigue, nausea/vomiting and cognitive functioning.
**Kassem et al.**	2019	Systematic Review	N = 2793 patients considered eligible from 14 studies	- Adverse Events	- CTCAE v4.0	- Systematic review reporting a rate of dysgeusia with Crizotinib and Alectinib ranging between 11-52%. - The most common AEs observed with ALK inhibitors were gastrointestinal toxicities. - There were differences between the toxicity patterns, with increased hepatic and gastrointestinal toxicities with Ceritinib, Crizotinib leading to more visual disorders, both Crizotinib and Alectinib causing more dysgeusia and Brigatinib causing more respiratory complications. -Low grade AEs were most commonly observed, and deaths related to treatment occurred in 0-1% of patients.
**Ueno et al. (** 36 **)**	2019	Prospective, Observational, Real-world data (Post-marketing surveillance: follow-up 52 weeks)	N = 2028 Japanese patients with ALK fusion gene-positive NSCLC treated with Crizotinib	- Adverse Events	- CTCAE v4.0	- Reported incidence rate of dysgeusia of 16.8%.
**Koizumi et al. (** 38 **)**	2015	Case report	–	–	–	- Case report on Grade 3 dysgeusia and anorexia, developing 5 days after starting treatment with Crizotinib. Toxicity completely regressed after switching to Alectinib. - Crizotinib is a multi-target receptor TKI for ALK, ROS1 and MET whereas, conversely, Alectinib targets ALK very selectively without activity against ROS1 and MET. -It remains unclear whether MET and ROS1 signals could be involved in oral tissues or cell impairment.
**Minakata et al. (** 33 **)**	2002	Case report	–	–	–	- Case report of severe gustatory disorder following the administration of cisplatin and etoposide CT combination. - It had been previously reported that cisplatin could lead to zinc displacement from its typical binding site, with gustin inactivation and subsequent hypogeusia. - Cisplatin-induced gustatory disorder could also be caused by disturbance to a peripheral nerve or receptor. The function of gustatory receptors could be suppressed either by cisplatin and/or etoposide. Some central nervous system dysfunction may also occur. - Gustatory disorders induced by anticancer agents usually reduce patients quality of life.

AEs. Adverse Events; CiTAS. Chemotherapy-induced Taste Alteration Scale; CT. Chemotherapy; CTCAE. Common Terminology Criteria for Adverse Events; EORTC QLQ-C30. European Organization for Research and Treatment of Cancer; ETOC. European Test of Olfatory Capabilities; HRQL. Health Related Quality of Life; NSCLC. Non-Small Cell Lung Cancer; RECIST. Response Evaluation Criteria in Solid Tumors; TAs. Taste Alterations; TSAs. Taste and Smell Alterations.

CT for lung cancer may involve a platinum-based compound and a third–generation agent, such as paclitaxel. Approximately two thirds of patients reported some type of dysgeusia when treated with paclitaxel-based CT (24).

In a study by Amézaga et al., CT with docetaxel led to the highest taste alteration scores in patient self-assessments (3). Steinbach et al. in Amézaga et al. found CT based on taxanes resulted in more severe taste disorders, particularly for the salt tastant. Metallic tastes, bad tastes in mouth and xerostomia have been reported in higher frequencies with paclitaxel, vinorelbine, anthracyclines and carboplatin. However, conclusions are limited by the use of a non-validated questionnaire to measure TAs. The exact mechanism causing metallic taste in patients receiving CT is unknown, but it can be generated from the CT substances being secreted in saliva and thus coming into contact with taste receptors (3).

Joussain et al. evaluated the olfactory performance of 15 lung cancer patients receiving cisplatin-based CT, concluding that whilst cisplatin did not influence odor identification and detection, a reduction in the pleasantness of food odors was evident, impairing food-related hedonic pleasure and ultimately, QoL (32).

Minakata et al. reported a case of severe gustatory disorder following the administration of a combination of cisplatin and etoposide CT (33). It had previously been reported by Henkin et al. in Minakata et al. (33) that cisplatin can displace zinc from its normal binding site and induce the inactivation of gustin, with consequent hypogeusia. Kanda et al. in Minakata et al. (33) hypothesized that cisplatin-induced gustatory disorder could be caused by disturbance to a receptor or a peripheral nerve. Cisplatin and/or etoposide could suppress gustatory receptor function, as well as leading to the occurrence of some dysfunction of the central nervous system (33).

Radiotherapy aims to destroy cancer cells by directly breaking the DNA helix strands, leading to cell death. Radiation to the chest affects the significantly radiosensitive epithelial cells lining the esophagus and pharynx. This can lead to radiation-induced side effects that can affect swallowing by disruption of the normal mucosal barrier and predisposition to fungal infection. Although typically resolving within 2 weeks after treatment conclusion, esophagitis and self-reported taste changes can continue to affect patients for weeks to months (34).

As the epidermal growth factor receptor (EGFR) is fundamental to epidermal and epithelial cells homeostasis, cutaneous or mucosal toxicities are commonly associated with the majority of anti-EGFR-treated patients (35).

Anaplastic lymphoma kinase (ALK) and ROS1 rearrangements also represent an established molecular alterations in a small subset of NSCLC (36).

Oral events induced by anti-EGFR TKIs are underreported compared to skin toxicities. Monotherapy with Erlotinib leads to an incidence of mucositis in patients of 8-20%, ranging between 17-24% with Gefitinib (35).

Crizotinib is a small-molecule, orally available tyrosine kinase inhibitor, that can suppress the activity of ALK and oncogene ROS1 kinases, causing cell cycle arrest at G1/S phase (37).

In a Crizotinib post-marketing surveillance performed in Japan, the incidence rate of dysgeusia was 16.8% (36).

A systematic review on ALK inhibitor studies, including Crizotinib and Alectinib, reported a rate of dysgeusia ranging between 11-52%. Despite none of them reporting high-grade dysgeusia, this might be a cause of patient uncompliance (37).

Qian et al. in Koizumi et al. (38) conducted a meta-analysis of Crizotinib published clinical trials and reported that the Crizotinib doses must be lowered or discontinued in 6.5% of patients due to toxicity. Koizumi et al. reported a case of G3 taste alteration and loss of appetite after 5 days of treatment with Crizotinib 250mg (twice daily), leading to a discontinuation of the drug. Toxicity completely regressed after switching to Alectinib 300mg (twice daily). It remains unclear whether dysgeusia could be dependent on the dose of Crizotinib. Whereas Crizotinib is a multi-target receptor TKI for ALK, MET and ROS1, Alectinib is highly selective for ALK without activity against MET and ROS1. It also remains unclear whether MET and ROS1 signals could be involved in oral tissues or cells impairment (38).

Moderate dysgeusia (grade 1 or 2) has been noted in fewer than 3% of PD-1 and PD-L1-treated patients (35).

#### Management and Treatment of Taste and Smell Alterations

Due to the increased risk of TSAs in lung cancer patients coupled with the related risks of experiencing weight loss and malnutrition, there is a high medical need for clinical trials focused on novel interventions to improve taste and smell. No guidelines for the treatment of smell and taste disorders are available, with general nutritional counselling that is offered to patients not earnestly addressing this subject (20). **Table 4** provides the summary of interventions to prevent or treat TSAs in lung cancer patients, included in the review.

**Table 4:** Summary of interventions to prevent or treat dysgeusia in cancer patients undergoing systemic treatment included in the review.

Authors	Year	Type of study		Sample size	Variables assessed	Method used to evaluate TSAs	Main results
**Nutritional Counseling**							
**Belqaid et al. (** 21)	2018	Qualitative Interview Study		N=17 lung cancer patients; 13 women, 4 men	- Patients behavior while experiencing treatment-related TSAs	- Qualitative Interview	- TSAs implied coming to terms with the presence of these symptoms and finding new personal strategies to overcome them - Health-care professionals’ involvement was generally described as limited; clinicians should be supported in providing more practical advice, normalizing information, and emotional support to better support patients manage TSAs.
**Taste and Smell Training**							
**Von Grundherr et al. (** 20)	2019	Phase II pilot trial, unicentric		N = 62 cancer patients undergoing CT (n=2 with lung cancer): - Intervention group (n=30): Taste and smell training + individual nutritional counseling - Non-intervention group (n=32): General nutritional information	- TSAs - Nutritional Status - HRQL	-”Taste Strips” method and “taste score” - MUST - EORTC QLQ-C30	- After 12 weeks, a clinically significant improvement of >2 points in the taste score were observed in 92% (n=23) of the intervention group patients, meeting the study’s primary endpoint. - Median QoL score did not significantly change in the intervention group at week 12 (p= 0.811), although a relevant clinical improvement was observed in 24% of patients (n=6).
**Schiffman et al. (** 41)	2007	Randomized Controlled Trial, unicentric		N=107 elderly cancer patients (n=95 with lung cancer) - Experimental group (n=54): Flavor enhancement products + Nutritional information - Control group (n=53): Nutritional information	- TSAs - Nutritional Status - QoL - Immune Parameters	- Taste and Smell Questionnaire, evaluation of taste and olfatory thresholds; - Mini Nutritional Assessment (MNA) - EORTC QLQ-C30 - Lymphocyte counts	- In the experimental group, MNA scores and physical function improved at the 8 months timepoint compared to the control group.
**Zinc Supplementation**							
**Hoppe et al. (** 42)	2021	Systematic Review	N=1120 patients undergoing cancer treatment from a total of 19 publications included. Types of cancer were not specified		Effect of Zinc Supplementation on: - Chemotherapy−induced mucositis -Radiotherapy−induced mucositis - Oral pain -Xerostomia - Dysgeusia	- Quantitative and qualitative methods	- Zinc supplementation revealed the occurrence, onset or severity of oral mucositis due to CT were not significantly affected by the intake of zinc; although positive effects on oral pain and severity and frequency of xerostomia were found. - For patients receiving RT or radio-chemotherapy, zinc had a significant impact on the onset, severity and duration of oral mucositis (except in nasopharyngeal carcinoma patients). - There was a common trend to taste improvement during radiotherapy, but not chemotherapy. - There was no observed impact on measured QoL, weight, fatigue, and survival.
**Fujii et al. (** 45)	2018	Retrospective, unicentric	N=634 patients (n=47 with lung cancer) receiving cancer CT - Patients with dysgeusia (n=80): Polaprezinc (150mg twice a day) until symptom disappearance - Patients without dysgeusia (n=554): Observation		- Grade 2 dysgeusia	- CTCAE v 4.0	- In patients who received oral Polaprezinc, the grade 2 dysgeusia’s 90 day recovery rates post symptom onset was 60%, significantly higher compared to the follow-up observation group (p=0.0007). - Grade 2 dysgeusia median recovery time was significantly lower in the Polaprezinc group than in patients in the follow-up group. - Polaprezinc was not as effective in elderly patients (≥65 years). - Pancreatic cancer patients were less responsive to Polaprezinc. - The patients with the highest Polaprezinc response were those suffering with colorectal cancer.
**Doi et al. (** 43)	2018	Review	-Cites 4 RCTs on the effect of zinc and Polaprezinc on the management of dysgeusia in cancer patients; only 2 involving lung cancer patients (Yamagata et al. being the only trial involving lung cancer patients exclusively; Lyckholm et al. involving 10 lung cancer patients from a sample of 41 patients with multiple cancer types)		-Taste Alterations	- Quantitative and qualitative methods	- Yamagata et al. explored the intravenous infusion of zinc during chemotherapy as a strategy for preventing taste disorders in patients receiving CT for lung cancer, with successful results. - Lyckholm et al. reported the failure of using oral zinc sulfate at 220 mg twice a day in improving CT-related taste alteration, loss or distortion of taste and smell, compared with placebo. - The studies demonstrated varied administration routes and dosing of zinc and Polaprezinc and included subjective forms of assessment of dysgeusia. Further studies with large samples using objective measures on taste testing should be conducted to support zinc use.
**Yamagata et al. (** 44)	2003	Randomized Controlled Trial, unicentric	N= 12 lung cancer patients under CT - Group A (n=7): CT + intravenous drip infusion containing zinc - Group B (n=5): CT + intravenous drip infusion without zinc		- Taste Acuity	- Electrogustometer (quantitative measurement)	- After 2 weeks of treatment, taste thresholds in all group B patients worsened at the corda tympani nerve area, whereas two thirds of patients in group A showed an improvement. Electrical taste thresholds significantly differed between both groups, after 2 and 4 weeks (p<0.05). - Although not significant, patients in group A revealed an improvement on the electrical taste thresholds in the glossopharyngeal nerve area, at 2 weeks. At 4 weeks, there wasn’t a significant difference in the taste characteristics in the glossopharyngeal nerve area between groups.
**Amifostine**							
**Komaki et al. (** 47)	2004	Randomized Controlled Trial, unicentric	N=62 patients with inoperable stage II or III NSCLC -Arm 1 (n=31): Chemoradiotherapy without Amifostine -Arm 2 (n=31): Chemoradiotherapy with Amifostine		- Toxicity from chemoradiotherapy - Survival outcomes	- NCI Common Toxicity Criteria	- Amifostine significantly reduced the rate of mild, moderate and severe esophageal toxicity (p=0.021), as well as the rate of severe pneumonitis (p=0.020) and neutropenic fever (p=0.046). - Mild hypotension (p<0.001), sneezing (p=0.039) and dysgeusia (p=0.029) were significantly more frequent in the Amifostine arm. - Amifostine had no apparent effect on survival.

CT, Chemotherapy; CTCAE, Common Terminology Criteria for Adverse Events; EORTC QLQ-C30, European Organization for Research and Treatment of Cancer; HRQL, Health Related Quality of Life; MNA, Mini Nutritional Assessment; NCI, National Cancer Institute; NSCLC, Non-Small Cell Lung Cancer; PZ, Polaprezinc; Pts, patients; QoL, Quality of Life; RECIST, Response Evaluation Criteria in Solid Tumours; TSAs, Taste and Smell Alterations; RT, Radiotherapy.

**Table 5:** Summary of the excluded articles with reasons.

References	Year	Study type	Reasons for exclusion
**Gift et al.**	2003	Retrospective, secondary analysis	Background article
**Turcott et al.**	2018	Abstract poster	Full article included in this review
**Lederhandler et al.**	2018	Case report	Wrong outcome (refers specifically to oral mucositis)
**Paule J.V. et al.**	2020	Prospective, longitudinal	Background article
**Catania et al.**	2020	Short Communication	Wrong outcome (refers specifically to dysgeusia and anosmia related to SARS-COV2 infection)
**Frowen J. et al.**	2020	Cross-sectional study	Background article
**Simeone et al.**	2019	Review article	Background article
**Van der Werf et al.**	2018	Pilot Study	Small sample and not exclusively related to lung cancer
**Sasaki et al.**	2017	Prospective study	Wrong outcome (non-physical concerns) and not exclusively related to lung cancer
**Wagland et al.**	2016	Cross sectional	Background article
**Thorne et al.**	2015	Review article	Background article
**Boltong et al.**	2012	Systematic review	Background article
**Watters et al.**	2011	Review	Background article
**Vadhan-Raj et al.**	2010	Randomized Controlled Trial	Wrong outcome (refers specifically to oral mucositis)
**Sanchez-Lara et al.**	2010	Cross-sectional study	Background article
**Ishinaga et al.**	2018	Cross-sectional study	Small sample and very specific to Japanese population

##### Nutritional Counselling

To ensure appropriate nutritional counselling, information related to possible taste changes that patients could experience should be made clear before starting treatment. This necessitates providing clinicians with evaluation measures that have been validated and ensuring they receive appropriate training in their use (12).

Belquaid et al. performed a qualitative interview to 17 lung cancer patients in order to understand what strategies or resources were used to deal with cancer-related TSAs. It was concluded that limited support from health-care professionals was provided, and that the majority of patients initiated management strategies by themselves, including coming to terms with TSAs, modifying their taste and smell experiences and finding emotional support in family and friends. As part of treatment, normalizing information about TSAs seemed to be important in promoting acceptance and adjustment to this reality, empowering patients to find their own solutions (21).

Maintaining protein intake is important for patients during treatment. Therefore, adding high protein foods into a patient’s diet should be encouraged, including eggs, dairy products, peanut butter, mild-tasting fish, chicken and soy meat substitutes (34).

Additionally, patients suffering from ageusia can try other techniques, such as flavoring meat, fish or chicken through sweet juice marination, sweet wine, and other sources including sweet-and-sour and Italian dressing. If nutritional supplements are too sweet, patients can try other options, including unflavored supplements or supplements that are based on juice or yogurt. CT-related metallic taste can be overcome by using plastic utensils, instead of metal ones (34).

For elderly patients or those suffering from umami hypogeusia, umami savoriness could help reduce any additional salt, sugar and fat consumption (24).

##### Taste and Smell Training

A clinical prospective pilot trial (the “TASTE trial”) assessed the possible short-term impacts of training taste and smell by applying the “Taste Strips Test” plus individual nutritional counseling to a group of cancer patients undergoing chemotherapy (<= or “CT”), 3% (n=2) having lung cancer. After 12 weeks, the study’s endpoint was met when a clinically significant improvement of >2 points was observed in 92% (n=23) of the intervention group patients (20).

A study by Schiffman et al. enrolled 107 cancer patients above 55 years old, the majority with lung cancer, to an experimental arm of flavor enhancement with aromas of actual foods plus nutritional counseling (n=54) and compared it with a control arm receiving nutritional information only (n=53). It was concluded that flavor enhancement plus nutritional counseling could improve patients’ nutritional status and QoL, suggesting that it is possible to minimize some chemosensory losses by providing cancer patients with the knowledge to improve the flavor of their foods (41).

##### Zinc Supplementation

Despite multiple investigations into how zinc can impact cancer treatment toxicities, there remains a lack of evidence to form a common consensus on its role (42).

To synthesize gustin, a salivary protein that is important in ensuring the integrity of taste buds, sufficient levels of zinc are required. Zinc deficiencies, therefore, can cause impaired taste and odor sensitivity. Maeda et al. in Cranganu *et al. (* 34) investigated the impact of taste alterations in 36 advanced lung cancer patients, and found higher taste abnormalities in patients with reduced serum zinc levels compared with the normal zinc level patient group (34).

A systemic review was performed by Hoppe et al. to evaluate the role of systemic zinc supplementation as complementary treatment for cancer patients (including 16 patients with lung cancer). Zinc was found to have no significant effects on chemotherapy-related oral mucositis or dysgeusia, although it seems to have positive effects on the prevention, severity and duration of RT or CT-induced oral xerostomia, mucositis and dysgeusia (42).

Polaprezinc is a chelating compound and anti-ulcer drug composed of a zinc ion, L-histidine, L-carnosine, and a β-alanine dipeptide. It has been observed to have antioxidant properties and to scavenge free radicals. Several preclinical and clinical studies showed its efficacy in reducing radiation-induced normal tissue damage. One review concluded that systemic Polaprezinc may be an acceptable option for reducing toxicities from chemoradiotherapy, being highly promising for preventing normal tissue damage in this context (43).

Yamagata et al. suggested that administering zinc intravenously during CT for lung cancer could help in preventing taste disorders and aiding patients in maintaining their QoL. Interestingly, a direct correlation between taste sensation and plasma zinc concentration could not be established, and the administration of zinc did not parallel an increase in the plasma zinc concentration (44).

A single-center retrospective study evaluated how zinc affects cancer patients’ taste disorders in which subjects with different types of cancer (including 47 patients with lung cancer) suffering from grade 2 CT-related taste disorders were given 150mg of zinc twice daily orally until symptoms disappeared, *vs* a placebo comparison group. The median recovery time was significantly lower in the group where patients received zinc (63 *vs* 112 days; p=0.019) (45).

##### Amifostine

Amifostine is a potent exogenous, free-radical scavenger and an established radioprotectant particularly for the prevention of radiation-induced xerostomia (43). It is currently the only US Food and Drug Administration (FDA) treatment for preventing moderate to severe radiation-induced xerostomia. Additionally, for patients with NSCLC or advanced ovarian cancer receiving repeated doses of cisplatin, it reduces the cumulative renal toxicity associated with treatment. Investigators have been interested in using this drug to prevent or reduce the severity of mucositis, but its effectiveness remains controversial (46).

One study examined the impact of amifostine on NSCLC patients receiving concurrent CT and RT and whether it impacted the acute toxicity associated with treatment. Interestingly, it demonstrated that in patients given amifostine, dysgeusia was more commonly present than in the control group patients (47).

## Discussion

From examining the literature, we believe no other reviews of dysgeusia and dysosmia in lung cancer patients have been conducted. TSAs are common in this particular population and may follow the diagnosis of the malignancy or develop during the course of cancer treatment, leading to a decrease in nutrient intake, weight loss, poor health-related quality of life (HRQoL) and worse disease prognosis.

TSAs’ intensity tends to change over time throughout the course of lung cancer treatment. Individual and contextual factors influence TSAs, including the presence of additional symptoms, side-effects of treatment and the overall life-situation of the patient (19).

Typically upon lung cancer diagnosis, a range of symptoms are present and can remain for the duration of the disease. These include weakness, fatigue, appetite and weight loss, nausea, vomiting and taste alterations (48).

On this basis, detection of these symptoms should warrant prompt assessment of chemosensory alterations (18).

In addition, it appears that the most predictive signal for the number of symptoms clustering in a patient is the stage of cancer the patient is experiencing (48).

Qualitative reports on TSAs changes are quite miscellaneous. The underlying mechanism for chemosensory alterations in cancer patients is not completely understood. This results from cancer population study heterogeneity, the multifactorial nature of odor and taste abnormalities, as well as the lack of standardized measurement tools for TSAs (3).

Taste disturbances seem to be present not only in cancer patients, but also, in patients suffering from inflammatory disorders, suggesting that taste perception deterioration can be linked to systemic inflammation inducing changes in interferon, toll-like receptor pathway and lipopolysaccharide, reducing taste progenitor cell proliferation and shortening taste bud cell lifespan (40).

For neoplastic disease, inflammation processes are induced by the release of multiple pro-inflammatory cytokines, which could be linked to the onset of taste disturbances. In this context, lung cancer may lead to changes in cell volume or osmolar content in central nervous system neurons, altering the sense of taste (22).

Upon observation of cryptogenic dysgeusia in lung cancer patients, it is necessary to consider the syndrome of inappropriate antidiuresis (SIAD), particularly if patients report unpleasantly sweet taste (27).

Interestingly, Kamoi et al. reported a case of a small cell lung cancer associated with hyponatremia, renal sodium loss and inappropriate antidiuresis unrelated to abnormal ADH plasma levels produced by the tumor. In this case, they were associated with an increased secretion of atrial natriuretic peptide (ANP) by the atrial tissue, resulting in a glomerular filtration rate increase and a decrease in tubular resorption of sodium (39).

TSAs may also develop as a consequence of CT, TKIs or RT-related side effects, through salivary gland damage and neuropathy, alteration of the structure of taste pores or conditioned aversions. A common complication of cytotoxic RT and/or CT is oral mucositis, being associated with dysgeusia, severe pain, odynophagia, malnutrition and dehydration. Contributing factors to smell and taste perception reductions include insufficient oral hygiene, xerostomia, older age, and alcohol or nicotine abuse. Other possible causes of unpleasant taste alterations arise from infection and gastrointestinal reflux leading to the production of extraneous substances (9).

In PD-1 and PD-L1-treated patients, moderate dysgeusia (grade 1 or 2) has also been observed in a minority of patients. One of the various immune-related adverse events to be aware of is pneumonitis (35). In the COVID-19 era, respiratory complaints, dysgeusia and anosmia are possible symptoms of SARS-CoV-2 viral infection, which can act as confounding factors in patients under immunotherapy when identifying treatment toxicities (49).

In lung cancer patients undergoing treatment with immune checkpoint inhibitors, the differential diagnosis between pulmonary toxicity induced by drugs, infective pneumonitis and tumor progression can be a major challenge (49).

Two randomized controlled trials (RCTs) investigated how the incidence and severity of oral side effects of cancer therapy, including dysgeusia, were affected by providing patients with dietary counseling and educational tapes. However, overall, dietary counseling as a single intervention only provided limited benefit to some patients, and the manner of delivery of the educational material to patients did not have a large impact (9).

On the other hand, nutritional counseling combined with taste and smell training and food enhancement may help the treatment of taste alterations before further problems or complications arise, particularly major weight loss and malnutrition (20, 40, 41).

Umami is regarded as the signal for protein-rich food and nutritious food, developing when meat and vegetables are cooked or roasted, resulting in the release of glutamate in food. Glutamate helps stimulate saliva production and appetite, reducing the craving for salt and sugar. A possible correlation between lower perception of umami and observed reductions in cancer patient meat consumption could exist. Testing of glutamate could be a predictor of a patient’s protein intake and its addition to a dish may increase savory perception. This has potential implications for supporting elderly cancer patients with healthier nutrition (40).

Despite conflicting results, studies reveal that systemic zinc supplementation or zinc-based solutions may help prevent and treat chemoradiotherapy-induced tissue damage, consequent mucositis and taste disorders (50).

In line with these data, Yanase et al. retrospectively evaluated the use of a 150 mg oral zinc solution 3 times per day before meals in patients with NSCLC under weekly administration of carboplatin and paclitaxel CT and concurrent chest RT. Grade ≥ 2 radiation esophagitis development was significantly delayed by oral zinc supplementation (HR, 0.397; 95% CI, 0.160–0.990; p = 0.047) at the point of reached cumulative radiation dose of 40Gy (51, 52).

Despite the fact that mucositis and xerostomia are frequently associated with taste alterations, we cannot necessarily assume that by treating the first we will be able to improve the latter, as it is reflected in various interventions improving oral mucositis, but not TSAs.

Many unanswered questions remain to fully understand the impact of lung cancer on taste and smell. Currently, standardized methods are unavailable to accurately and consistently measure taste and smell dysfunction across different clinical settings. This makes it more difficult to analyze and interpret study results given that self-reported taste and smell function is often not objective. Without understanding taste and smell tissue development, regeneration and degeneration at a cellular level, it is not possible to identify and develop treatments to target the sources of sensory dysfunction. For example, a consistent method for regrowing human taste receptor cells or olfactory neurons after injury or illness remains elusive, nor a method for reconnecting those cells to the areas of the brain responsible for taste and smell perception. Also, little progress has been made in analyzing the fluids specific to each type of tissue (saliva for taste, mucus for smell) for inflammation-related biomarkers or cellular dysfunction (4).

So far, no guidelines for the treatment of taste disorders are available, while there is a large need for trials to improve cancer patient’s smell and taste with alternative interventions, as dietary counseling alone seems to be of modest benefit to some patients (9).

## Conclusions

Given the high frequency of TSAs and their impact on nutritional status, treatment tolerance and QoL, normalizing and promoting adequate adjustment to TSAs in lung cancer patients is relevant. Potential taste and smell changes prior to lung cancer treatment should be communicated clearly to patients, requiring clinicians to be appropriately trained and provided with validated evaluation measures. Food enhancement through taste and smell training plus personalized nutritional counseling combined with zinc supplementation or use of oral zinc-based solutions seem to be helpful in preventing and treating mucositis and taste disorders in lung cancer patients under chemo-radiation treatment, although adequately powered prospective RCTs are still lacking. Overall, more work is needed to compensate for the lack of methods to standardize smell and taste dysfunction measurement across different clinical settings and the lack of understanding of the increased risk of taste alterations associated with some patient and clinical characteristics. Finally, it is critical to better understand, at the cellular level, the development and regeneration of smell and taste tissues. This could significantly help identify the sources of sensory dysfunction and support the creation of targeted strategies for treating taste and smell disorders in this cancer population.

## Data Availability Statement

The original contributions presented in the study are included in the article/supplementary material. Further inquiries can be directed to the corresponding author.

## Author Contributions

All authors made substantial contribution for the writing of this systematic review. PR inspired and encouraged the first author to review this topic. ASS and DSD were responsible for systematically reviewing the literature ensuring an independent selection of all the papers included. ASS was the main responsible for the conception, planning and writing of the first draft. MLC provided major support in coordinating and structuring the manuscript itself. PR, AM and FP helped to comprehensively and critically write the final draft; together with TS and PMN, who analyzed the manuscript, rectifying the language and validating the integrity and structure of the content included in this review. All authors contributed to the article and approved the submitted version.

## Conflict of Interest

Author FP was employed by company BlueClinical.

The remaining authors declare that the research was conducted in the absence of any commercial or financial relationships that could be construed as a potential conflict of interest.

## Publisher’s Note

All claims expressed in this article are solely those of the authors and do not necessarily represent those of their affiliated organizations, or those of the publisher, the editors and the reviewers. Any product that may be evaluated in this article, or claim that may be made by its manufacturer, is not guaranteed or endorsed by the publisher.
